# Recognizing and Responding to Elder Mistreatment: Developing and Testing an Online Training for EMS Practitioners

**DOI:** 10.1093/geroni/igab046.3013

**Published:** 2021-12-17

**Authors:** Kristin Lees Haggerty, Dana Wardlaw, Melanie Miller, Randi Campetti, Athi Myint-U, Brad Cannell

**Affiliations:** 1 Education Development Center, West Roxbury, Massachusetts, United States; 2 Education Development Center, Waltham, Massachusetts, United States; 3 Education Development Center, Jamaica Plain, Massachusetts, United States; 4 Education Development Center, Education Development Center, Massachusetts, United States; 5 University of Texas Health Science Center at Houston, Dallas, Texas, United States

## Abstract

Elder mistreatment is an urgent and under recognized public health concern with devastating consequences for older adults, families, and health systems. Risk for elder mistreatment has increased during the COVID-19 pandemic, further highlighting the urgency to address it. Prehospital emergency medical service (EMS) practitioners have unique opportunities to recognize signs of elder mistreatment but often lack the training and tools required to facilitate consistent identification and intervention. To address this gap, Education Development Center collaborated with a team of expert advisors and EMS practitioners to develop and pilot test Recognizing and Responding to Elder Mistreatment: An Online Training for EMS Practitioners with funding from the RRF Foundation for Aging. This training aims to prepare EMS practitioners to recognize potential mistreatment and report suspected elder mistreatment in line with state laws and their professional code of ethics. In this presentation, we will describe the iterative development process, present results from a pilot test conducted with EMS practitioners in Massachusetts and share strategies and progress for disseminating the training nationally. The pilot study utilized a pre-post design to assess changes in knowledge, attitudes, and practices at baseline, immediately after and two months following participation in the training. Results indicate statistically significant improvements in knowledge related to elder mistreatment identification and response from pre- to post-training and maintenance of these improvements two months later. Participants reported feeling more prepared to address elder mistreatment in their work as EMS practitioners and applying their new knowledge and skills during the two months following the training.

